# Brain MRI Pattern Recognition in Neurodegeneration With Brain Iron Accumulation

**DOI:** 10.3389/fneur.2020.01024

**Published:** 2020-09-10

**Authors:** Jae-Hyeok Lee, Ji Young Yun, Allison Gregory, Penelope Hogarth, Susan J. Hayflick

**Affiliations:** ^1^Department of Neurology, Research Institute for Convergence of Biomedical Science and Technology, Pusan National University Yangsan Hospital, Pusan National University School of Medicine, Yangsan-si, South Korea; ^2^Department of Neurology, Ewha Womans University Seoul Hospital, Ewha Womans University College of Medicine, Seoul, South Korea; ^3^Departments of Molecular and Medical Genetics, Oregon Health and Science University, Portland, OR, United States

**Keywords:** neurodegeneration, iron, NBIA, magnetic resonance imaging, pattern

## Abstract

Most neurodegeneration with brain iron accumulation (NBIA) disorders can be distinguished by identifying characteristic changes on magnetic resonance imaging (MRI) in combination with clinical findings. However, a significant number of patients with an NBIA disorder confirmed by genetic testing have MRI features that are atypical for their specific disease. The appearance of specific MRI patterns depends on the stage of the disease and the patient's age at evaluation. MRI interpretation can be challenging because of heterogeneously acquired MRI datasets, individual interpreter bias, and lack of quantitative data. Therefore, optimal acquisition and interpretation of MRI data are needed to better define MRI phenotypes in NBIA disorders. The stepwise approach outlined here may help to identify NBIA disorders and delineate the natural course of MRI-identified changes.

## Introduction

Neurodegeneration with brain iron accumulation (NBIA) is a group of inherited disorders with hallmark features that include abnormal iron accumulation in the basal ganglia, mainly the globus pallidus (GP) and substantia nigra (SN) (1). Ten associated genes have been identified [Table 1; (2)]. The four most common NBIA disorders include pantothenate kinase-associated neurodegeneration (PKAN), phospholipase A_2_-associated neurodegeneration (PLAN), mitochondrial membrane protein-associated neurodegeneration (MPAN), and beta-propeller protein-associated neurodegeneration (BPAN) (3). Recently, new candidate genes have been described with the advent of next-generation sequencing (4). However, the scarcity of cases makes it impossible to determine their relevance to NBIA disorders (5).

**Table 1:** MRI characteristics of NBIA subtypes.

**Disease**	**Gene/** **inheritance**	**Iron distribution**	**Core features**	**Early features**	**Additional features**
PKAN	*PANK2*/AR	GP, SN, STN	Eye-of-the-tiger sign in GP (typically, the surrounding T2 hypointensity)	Linear T2 hyperintense streak along the medial border of GP in infancy [10] Isolated hyperintense center during early childhood [10]	Basal ganglia calcification
MPAN	*C19orf12*/AR, AD	GP, SN	Preserved isointense signal in medial medullary lamina of GP	Three signal layers in GP (T2*WI) T2 hyperintense “dot” in the central part of GP [9]	Diffuse brain atrophy Variable degree of WM involvement
BPAN	*WDR45*/XD	SN, GP	Halo sign in SN	Thin corpus callosum Myelination delay T2 hyperintensity and swelling in SN, GP, deep cerebellar nuclei [41] T2 (or SWI) hypointensity, predominantly in SN and/or T1 hyperintensity	Diffuse brain atrophy Basal ganglia calcification Variable degree of WM involvement
PLAN	*PLA2G6*/AR	GP, SN	Disproportionate cerebellar atrophy and/or cerebellar cortical hyperintensity	Vertically oriented thin corpus callosum [54] Hypertrophy of the clavum [71]	Supratentorial atrophy Variable degree of WM involvement Hypoplastic optic tracts and chiasms
FAHN	*FA2H*/AR	GP, SN	WM hyperintensities (periventricular, parietal predominance) Pontocerebellar atrophy Thin corpus callosum		Supratentorial atrophy
Neuroferritinopathy	*FTL*/AD	Widespread, basal ganglia, thalamus, cerebellum, cerebral cortex	Cavitation involving GP and putamen	SWI hypointensity in GP, SN, thalamus, red nucleus, and dentate nucleus, without cavitation, and cortical pencil lining sign [45]	Diffuse brain atrophy WM hyperintensities
Aceruloplaminemia	*CP*/AR	Widespread, uniform basal ganglia, thalamus, cerebellum, cerebral cortex	Iron accumulation in the brain, liver, pancreas, and myocardium		Diffuse brain atrophy WM hyperintensities
Woodhouse-Sakati syndrome	*DCAF17*/AR	GP, SN	WM hyperintensities (frontoparietal/ periventricular WM)		Small pituitary gland
Kufor-Rakeb syndrome	*ATP13A2*/AR	Putamen, caudate, GP			Diffuse brain atrophy
CoPAN	*COASY*/AR	GP, SN			T2 hyperintensity of caudate, putamen, and thalamus Swollen putamen and caudate GP calcification

*PKAN, pantothenate kinase-associated neurodegeneration; MPAN, mitochondrial membrane protein-associated neurodegeneration; BPAN, beta-propeller protein-associated neurodegeneration; PLAN, PLA2G6-associated neurodegeneration; FAHN, fatty acid hydroxylase-associated neurodegeneration; CoPAN, COASY protein-associated neurodegeneration; AR, autosomal recessive; AD, autosomal dominant; XD, X-linked dominant; GP, globus pallidus; SN, substantia nigra; WM, white matter*.

Evidence by magnetic resonance imaging (MRI) of excessive brain iron indicates the possibility of NBIA. One established hallmark MRI feature of NBIA is the presence of T2 hypointense lesions in the GP and SN on T2-weighted images (T2WI) (3, 6). Certain MRI abnormalities may help distinguish among the NBIA disorders and facilitate more definitive diagnosis (1, 7). However, mutations in NBIA-related genes may not always lead to pronounced iron deposition (1, 7, 8). A significant number of patients confirmed to have an NBIA disorder by genetic testing have MRI features that are atypical for their specific disease (7, 9, 10). The appearance of specific MRI patterns depends on the stage of the disease and the patient's age at evaluation (1, 10), and evidence for iron accumulation may be absent or subtle early in the disease course. This phenomenon is particularly common in younger patients, where whole exome sequencing often leads to early diagnosis (3). Minor lesions visible in the early stages of disease and more extensive lesions in the late stages often are non-specific.

MRI interpretation can be challenging in rare brain diseases and can be limited by heterogeneously acquired MRI datasets, individual interpreter bias, and a lack of quantitative and longitudinal data. Therefore, optimal acquisition and interpretation of MRI data are needed to better define MRI phenotypes in the NBIA disorders. We describe here the evolution of MRI characteristics and provide a practical approach to identify NBIA subtypes.

## Identification of Iron-Specific Basal Ganglia T2 Hypointensity

A routine brain MRI, without iron-sensitive sequences, is often suboptimal for evaluating for a possible NBIA disorder (7). Iron-sensitive sequences, especially susceptibility weighted imaging (SWI) and T2^*^-weighted imaging (T2^*^WI), can more clearly depict the increase and extent of iron deposition, even in small gray matter nuclei (10, 11). High-field strength MRI can detect iron with improved sensitivity (12).

To correctly diagnose abnormal brain iron accumulation, the interpreting physician should have a working knowledge of normal age-dependent signal hypointensities on MRI (7). The GP and SN normally become hypointense on T2WI around the end of the first decade of life when compared with signal in the adjacent normal-appearing white matter (13). Iron concentration in the basal ganglia increases with age. An “internal signal-intensity reference” that we empirically use to determine if iron is indeed increased over “normal” is to compare the GP and SN to the red nucleus (RN). They normally appear slightly more hypointense relative to the RN based on their higher iron content at all ages (14). If the signal in GP or SN is significantly more hypointense than in RN, then there is likely to be increased iron. To correct for inconsistencies in the reference standard, signal hypointensity can also be normalized by dividing the structure signal intensity by the mean signal intensity of the cerebrospinal fluid (15, 16).

Once T2 hypointensity is identified, iron-sensitive sequences should be reviewed to distinguish excessive iron deposition from other causes of T2 hypointensity. Hypointense basal ganglia have been observed in hypomyelinating leukodystrophy, lysosomal storage disorders, and other metal accumulation disorders (17). Due to the paramagnetic property of iron, the degree of signal loss is profoundly enhanced in iron-sensitive sequences. Manganese is also paramagnetic, and its deposition typically causes high signal intensity on T1WI (11). Although iron has a T1-shortening effect that can appear as high signal intensity on T1WI, the degree of T1 hyperintensity is variable and is influenced by the status of iron and T1WI parameters (11). Computed tomography (CT) scans may be more useful than MRI in differentiating calcifications from iron deposits. Basal ganglia calcification can co-exist with iron accumulation in NBIA cases, although its frequency is unknown (18–21).

## Regional Distribution of Excessive Iron Accumulation

In most forms of NBIA, excessive iron deposition is mainly confined to the GP and SN (Table 1). Other iron-rich deep nuclei in the gray matter, like the dentate nucleus, can occasionally be affected to a lesser extent but only in specific NBIA disorders (22).

In PKAN, iron-related hypointense signals on SWI are restricted to the GP, SN, and subthalamic nucleus (STN) and the fiber tracts between these structures (10). In BPAN, the earliest and most intense iron deposition occurs in the SN as opposed to the GP, unlike PKAN and other NBIA disorders (7). Widespread brain iron accumulation involving the basal ganglia nuclei, thalami, dentate nuclei, and cerebral and cerebellar cortices may develop in aceruloplasminemia and neuroferritinopathy (7, 22). The symptom onset for both of these diseases has been described in adults (1). All basal ganglia and thalami are more uniformly involved in aceruloplasminemia (22). Cortical iron deposition appears as thin hypointense lines on SWI, referred to as pencil-lining in neuroferritinopathy (23, 24). Even in an asymptomatic mutation carrier for this autosomal dominant disease, a characteristic pattern of iron deposition can be seen on iron-sensitive sequences (25). Of the limited number of patients reported, only a small portion of cases with Kufor-Rakeb syndrome had iron accumulation within the putamen and caudate nuclei (26, 27).

## Evolution of Pallidonigral Abnormalities

The characteristic pallidonigral lesions of the major NBIA disorders are established diagnostic clues (Table 1). These include the “eye-of-the-tiger” sign in PKAN, preservation of isointense signal in the medial medullary lamina in the GP of MPAN patients, the “halo” sign in the SN of BPAN patients, and “cavitation” in neuroferritinopathy. Other NBIA disorders do not have distinct pallidonigral lesions. However, the morphological patterns of pallidonigral lesions can vary according to the patient's age. Typical findings may not appear until adolescence or early adulthood and are therefore not usually useful in infancy or early childhood. For these reasons, the frequency of MRI clues varied across studies.

**Figure 1:**
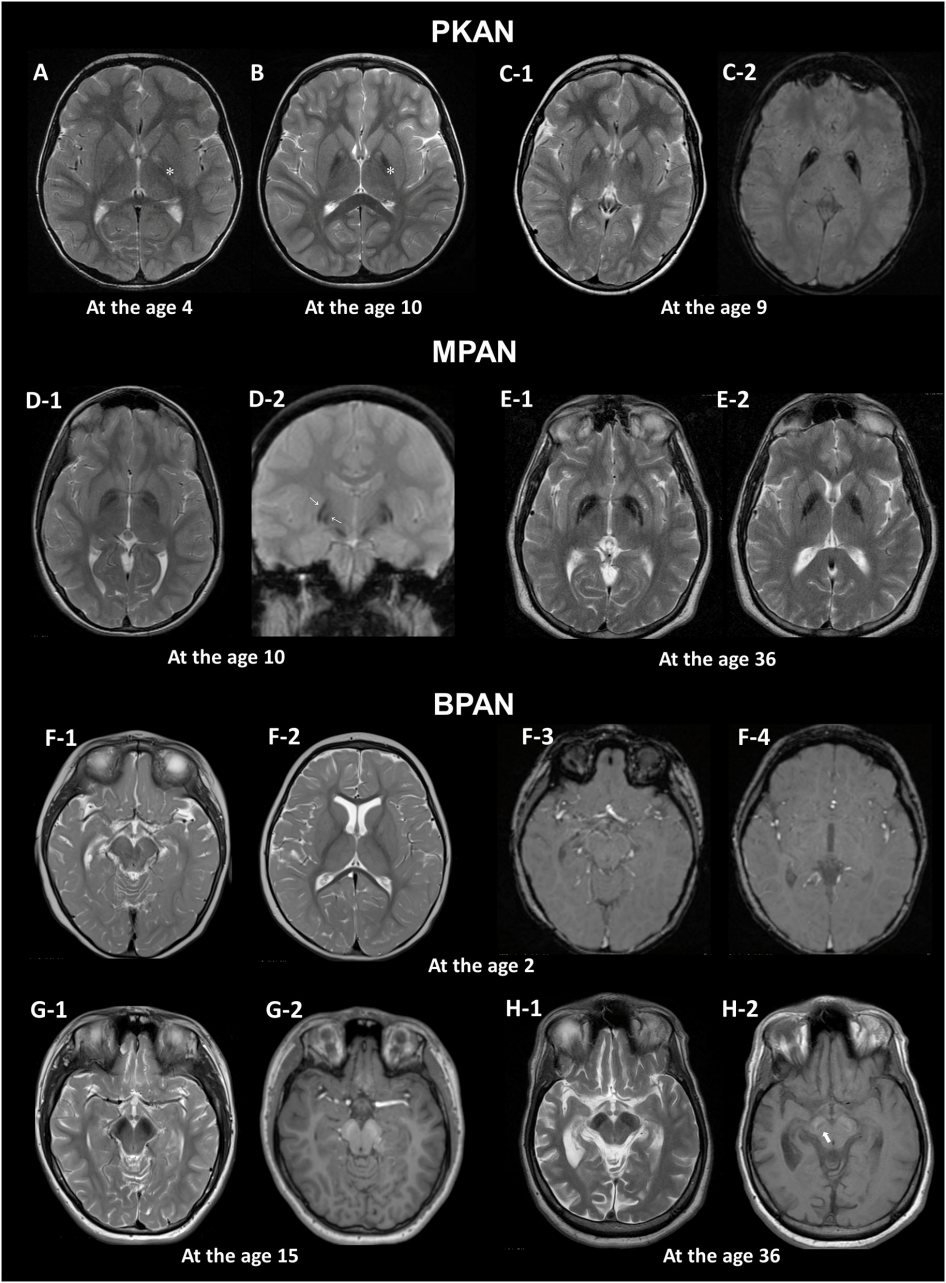
MRI hallmarks of PKAN **(A–C)**, MPAN **(D,E)**, and BPAN **(F–H)**. In a PKAN patient with serial MRIs, the surrounding T2 hypointensity decreases in signal intensity with age (**A,B**, asterix). The PKAN-specific eye-of-the-tiger sign at the level of the anterior commissure **(C-1)** is better defined by the region-specific pattern of iron deposition on susceptibility-weighted image **(C-2)**. In a younger patient with MPAN, T2- and T2*-weighted images shows preservation of isointense signal in the middle of inner and outer layers of iron accumulation (**D-1, D-2**, thin arrows). The typical streaking of the medial medullary lamina on T2-weighted image becomes more pronounced as signal intensity in the GP decreases, which is typically visible at the level of the anterior commissure **(D-1, E-1)**. Brain MRI scans of three BPAN patients at different ages show age-related MRI changes. Brain MRI of a patient with BPAN at the age of 2 shows no abnormality on T2-weighted **(F-1, F-2)** and susceptibility-weighted **(F-3, F-4)** images. The substantia nigra is seen as hypointense on T2-weighted image **(G-1, H-1)** and hyperintense on T1-weighted image, whereas the central hypointense band is not demonstrated **(G-2)**. T1-weighted image demonstrates hyperintensity of the substantia nigra and cerebral peduncle surrounding a central linear band of hypointensity, also known as the halo sign (**H-2**, thick arrow).

The detection of these specific MRI signs can also be influenced by MRI acquisition settings (10). Image planes and levels for optimal visualization should be selected for efficient identification of the signs (Figure 1).

### Eye-of-the-tiger Sign in PKAN

The hallmark of PKAN is the eye-of-the-tiger sign that comprises a round hyperintense center and surrounding hypointensity in the GP on T2WI. This sign in its classic form is not observed in other NBIA disorders, including in patients with *COASY* protein-associated neurodegeneration (CoPAN), which affects coenzyme A metabolism, similar to PKAN (21, 28).

The earliest change observed in infancy was the linear hyperintense streak along the medial border of the GP. During early childhood, T2WI mostly shows the isolated hyperintense center in the anteromedial region, which is typically visible at the level of the anterior commissure (29). The surrounding T2 hypointensity tends to increase in size and decrease in signal intensity with age (Figures 1A,B). Signal hypointensity on SWI was first detected in a 3-year-old patient, and advanced from the medial to the lateral portion of the GP (10, 18). In adult patients, the hyperintense center varies in shape, from small and round to streaked, or is lost entirely. Furthermore, the T2 hyperintense area of the sign may be obscured by diamagnetic calcium deposits (10, 18).

This sign has been reported to occur as an imaging phenocopy in other conditions, such as carbon monoxide intoxication, neuroferritinopathy, MPAN, Wilson's disease, multiple system atrophy, as well as in healthy adults (10, 30–32). The combination of T2 hypointense and hyperintense lesions, resulting from iron deposition and gliosis, respectively, can mimic the eye-of-the-tiger sign. The PKAN-specific eye-of-the-tiger sign is better defined by the region-specific pattern of iron deposition on SWI (10).

### Preserved Isointense Signal in Medial Medullary Lamina in MPAN

Iron accumulation in the GP, followed by the SN, is evident on MRI (1). A characteristic feature in MPAN is T2 iso- to hyperintense streaking in the region of the medial medullary lamina between the abnormally hypointense GP interna and externa (33, 34), typically visible at the level of the anterior commissure (Figure 1). However, this MRI finding is present only in some MPAN patients (33, 35, 36). In recent reports, this sign was detectable in about half of patients and brain MRI may be normal in the early stages of MPAN (9, 37, 38).

Typical linear streaking (Figure 1E) develops over time and becomes more pronounced as signal intensity in the GP decreases due to iron accumulation. Initially, the GP appears as three signal layers consisting of the isointense signal layer in the middle of the hypointense inner and outer signal layers of iron accumulation, and is more contrasted on iron-sensitive T2^*^WI (Figure 1D).

### Substantia Nigra Halo Sign in BPAN

A unique feature of BPAN is the presence of a hyperintense halo surrounding a central band of hypointensity on axial T1WI within the SN [Figure 1H; (19, 39, 40)]. T1 hyperintensity extends to the cerebral peduncles. T2WI shows prominent hypointense signal in the SN and cerebral peduncles. The SN is more hypointense relative to the GP, indicating higher levels of iron.

MRI changes in BPAN develop as age-dependent phenomena (Figures 1F–H). A serial MRI study demonstrated that SWI hypointensity in the GP and SN was observed after 2–7 years old, whereas T2 hypointensity after 4–7 years old (41). Here, the T1 hyperintense signal in the SN was detectable by early in the second decade of life (40, 42, 43). A characteristic halo sign appears later in the disease course, particularly as parkinsonism becomes evident in early adulthood (1).

### Cavitation in Neuroferritinopathy

Cavitation involving the GP and putamen is unique to neuroferritinopathy among the NBIA disorders. A neuropathological study has demonstrated fluid accumulation within these cysts (22). Cavitary lesions with T2 hyperintensity are lined by hypointense rims secondary to prominent iron deposition (23). In a case report, Fluid-attenuated inversion recovery (FLAIR) images exhibited a tri-lamellar intensity consisting of an outer layer with iron deposition, a middle layer with gliosis, and a cystic core (44). This may represent different stages of expanding pathology. Cavitary lesions occur late in the disease, usually after excessive iron deposition, and evolve with time (22, 45).

## Non-iron and Extrapallidal Abnormalities

Besides excessive iron, extrapallidal MRI abnormalities are helpful to facilitate diagnoses [Table 1; (7, 17)]. Neuroradiographic anatomic regions where non-iron and extrapallidal abnormalities are common in the NBIA disorders are summarized in Supplementary Figure 1.

### The Extent and Magnitude of the Cerebral Atrophy

The extent of atrophy may depend on the nature of the underlying pathology (8, 46). Widespread α-synuclein-positive Lewy pathology has been identified in PLAN and MPAN (47, 48). Tau-positive neurofibrillary tangles are common in the brains of patients with BPAN (49). Indeed, pathologic α-synuclein and tau can spread extensively across the brain using a prion-like mechanism of propagation (50). Therefore, neurodegenerative changes can be more widely distributed throughout the brain. Serial MRI studies showed that brain atrophy progresses with the disease course (9, 34). On the contrary, in PKAN, neuronal loss, and astrogliosis are largely restricted to the GP in the absence of misfolded protein aggregates (49).

Visual rating scales are useful tools in assessing the severity of atrophy objectively. An established 4-point rating scale (51–53) is applicable for the assessment of cerebral and cerebellar cortical atrophy in NBIA disorders. In addition, planimetric analysis using sagittal T1WI can be used to evaluate volumetric changes in midsagittal structures including the corpus callosum, cerebellar vermis, and brainstem.

### Disproportionate Cerebellar Atrophy in PLAN

In the majority of NBIA disorders, brain atrophy, if present, is usually generalized and has been commonly described without regional dominance. Cerebellar atrophy is a hallmark feature in PLAN, and is often the earliest sign on MRI (7, 54). It has been seen in up to 95% of patients with *PLA2G6* mutations (55). In infantile neuroaxonal dystrophy (NAD) and childhood-onset PLAN (juvenile NAD), cerebellar atrophy is a near universal feature (56, 57). Patients with an earlier disease onset showed a more severe cerebellar atrophy, which was assessed using the ratio of the mid-sagittal vermis size over the total posterior cranial fossa size (57). T2 or FLAIR hyperintensity in the cerebellar cortex often accompanies cerebellar atrophy (54, 58). In contrast, excessive iron deposition in the GP is seen in only up to half of PLAN cases (7, 55). Disproportionate cerebellar atrophy and iron deposition can be absent in adult-onset PLAN (56, 59, 60), where there may be only frontally predominant atrophy (61) and MRI may even appear normal (56).

### Thin Corpus Callosum

Thinning of the corpus callosum is a uniform feature in fatty acid hydroxylase-associated neurodegeneration (FAHN) (7, 62). Abnormal posterior corpus callosum that are thin and vertically oriented were noted in some cases of PLAN (54). Corpus callosum thinning may be an early sign of BPAN in the absence of excessive iron during infancy and early childhood (39, 40, 63).

It is important to evaluate the thickness and the morphology of corpus callosum in association with other findings observed in NBIA disorders, such as cerebral atrophy, myelination defect, or white matter damage (64).

### White Matter Hyperintensities

T2 hyperintensities in white matter have been reported in most NBIA subtypes (1, 7, 8, 17) and are prominent in FAHN, Woodhouse-Sakati syndrome (WSS), and aceruloplasminemia. In a large cohort of patients with FAHN, the most common findings were white matter changes (100%), followed by ponto-cerebellar atrophy, GP hypointensity, and thin corpus callosum (62). T2 hyperintense white matter abnormalities were consistently found in the periventricular white matter with parietal predominance. In WSS, frontoparietal and periventricular white matter lesions were the most common non-iron abnormalities (65). And, older age was associated with a more severe degree of white matter lesions. In this study, the extent of white matter lesions was graded as none, mild (small focal), moderate (patchy scattered), or severe (diffuse confluent) according to their site, shape, confluency, and multifocality (65, 66). Prominent white matter hyperintensity is frequently noted in aceruloplasminemia (7). White matter hyperintensities in the posterior frontal and parieto-occipital regions extend caudally to the brainstem along the corticospinal tracts in a patient with aceruloplasminemia (67). Confluent T2 hyperintensities in white matter, localized mostly to the periventricular region, may be observed to a lesser extent in MPAN, BPAN, PLAN, and neuroferritinopathy (7–9, 40).

Although white matter T2 hyperintensity may be observed in NBIA disorders, diffuse cerebral hypomyelination is generally not a feature of these disorders (17). Delayed myelination has been reported in some cases of BPAN diagnosed in infancy and childhood (63, 68). However, the MRI findings in these cases were not described in enough detail to assess myelination. Hypomyelination is defined as an unchanged pattern of deficient myelination on two successive MRI scans at least 6 months apart in a child older than 1 year (69, 70). Myelinated white matter structures have a higher signal than do gray matter structures on T1WI and a lower signal on T2WI (69).

### Miscellaneous Findings

Apparent hypertrophy of the clavum has been proposed as an important early feature of PLAN and may precede cerebellar atrophy (57, 71). Confirmation of clavum enlargement was made by comparison of its largest anteroposterior dimension on mid-sagittal T1WI with age-matched controls (71). Hypoplastic optic tracts and chiasms are seen in infantile NAD (72). SN swelling in the absence of iron deposition has been described as an early sign of BPAN (73), although the reason for SN enlargement is unknown. In a serial MRI study, transient T2 hyperintensity and swelling in the SN, GP, and/or deep cerebellar nuclei was observed during the episodes of pyrexia and seizures (41). Similarly, swelling and T2 hyperintensity of the caudate nucleus, putamen, and thalamus have been found in CoPAN (28). Small pituitary glands are common MRI abnormalities in WSS (65).

## Current Limitations and Future Directions

Visual inspection of MRI can be highly subjective, and results can be varied. Objective interpretation is limited due to a lack of consistent methods to quantify the severity of MRI findings. A feasible way to objectively assess MRI severity is to use established visual rating scales or planimetric analysis as described above.

Iron quantification can be challenging, particularly within routine clinical settings. T2WI and SWI do not directly reflect iron concentrations (74). Instead, both the transverse relaxation rate (R2^*^) and quantitative susceptibility mapping are highly sensitive and accurate for measuring iron content in the brain (75). In a recent randomized trial of deferiprone for PKAN, iron concentrations in the GP were measured by MRI-R2^*^ mapping (76). Diffusion-tensor imaging study demonstrated a significant increase of FA in patients with PKAN suggest the presence of abnormal iron in deep gray matter nuclei, even in the absence of its demonstration on T2^*^WI (77). An optimized protocol for quantitative MRI analysis is needed to monitor disease progression and treatment response.

Finally, studies that have investigated the pathological correlates of MRI signal alterations are rare. In some NBIA disorders, excessive iron deposition has not yet been demonstrated pathologically due to lack of autopsy-proven cases. Further research is needed to verify the exact underlying pathology of MRI abnormalities.

## Conclusion

Specific NBIA disorders can be diagnosed by identifying characteristic MRI changes in combination with clinical findings. If an NBIA disorder is suspected or in the differential diagnosis, then iron-sensitive sequences should be included in an initial MRI. It is important to consider that MRI features specific to each NBIA disorder develop in an age-dependent manner and evolve during the disease course. The stepwise pattern-recognition approach outlined here may help to distinguish specific NBIA disorders as well as to delineate the natural course of MRI changes.

## Author Contributions

J-HL and SH: design and conceptualization of the study, analysis and interpretation of the data, drafting the manuscript, and final approval of the manuscript. PH: design and conceptualization of the study and final approval of the manuscript. AG and JY: acquisition of the data and final approval of the manuscript. All authors reviewed the manuscript.

## Conflict of Interest

The authors declare that the research was conducted in the absence of any commercial or financial relationships that could be construed as a potential conflict of interest.

## Supplementary Material

Supplementary Figure 1 Neuroradiographic anatomic regions where non-iron and extrapallidal abnormalities are common in the NBIA disorders. A normal MRI is used to show structures. The specific NBIA disorders are listed under each abnormality.
