# Supplementary figures and images for: Brain MRI Pattern Recognition in Neurodegeneration With Brain Iron Accumulation
Source: Front Neurol. 2020 Sep 10;11:1024. doi: 10.3389/fneur.2020.01024 (PMC7511538; doi:10.3389/fneur.2020.01024)

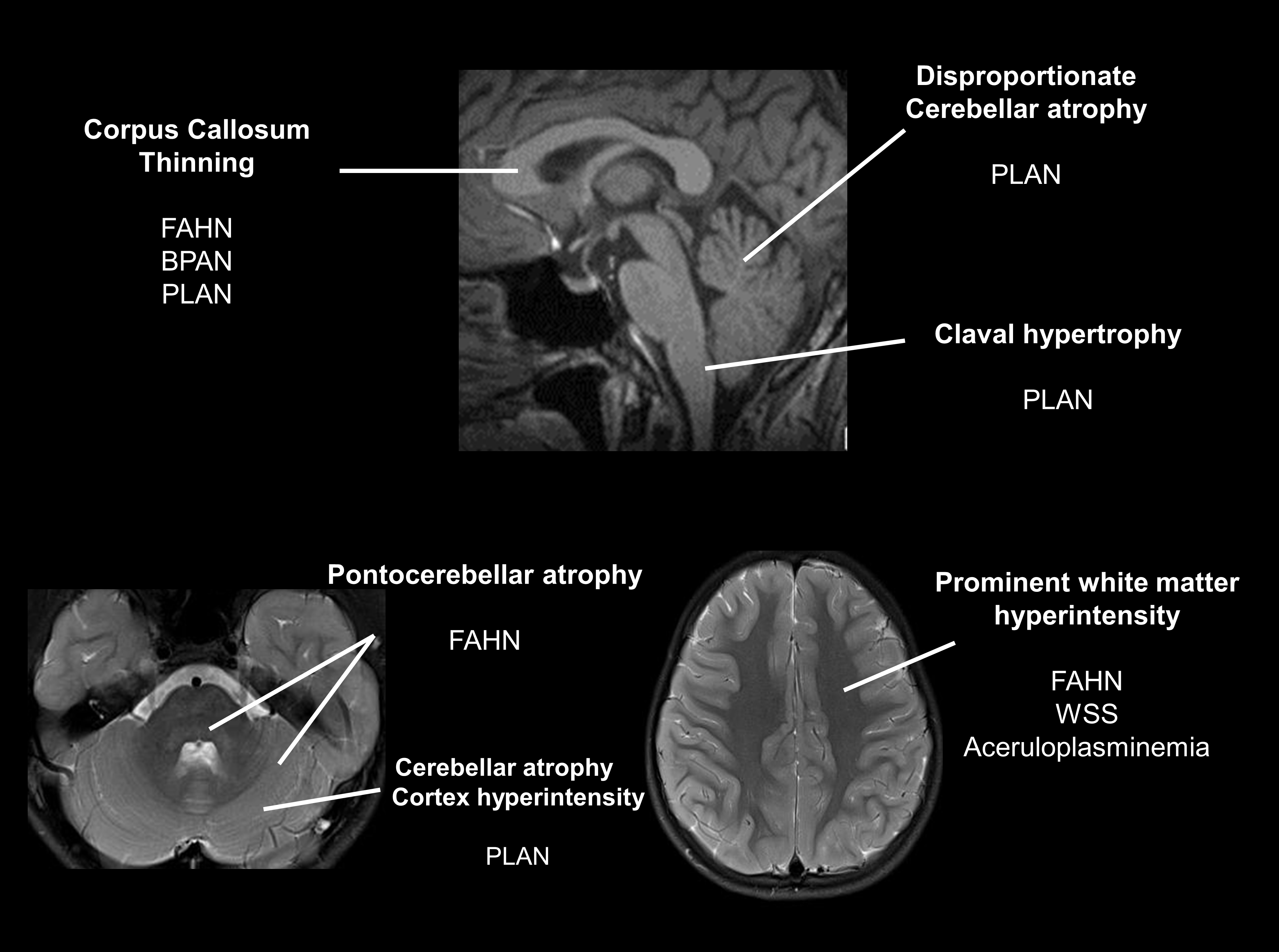

Supplement: Supplementary Figure 1 — Neuroradiographic anatomic regions where non-iron and extrapallidal abnormalities are common in the NBIA disorders. A normal MRI is used to show structures. The specific NBIA disorders are listed under each abnormality. [file Image_1.TIF]
